# *twoaxistracking* – a python package for simulating self-shading of two-axis tracking solar collectors

**DOI:** 10.1016/j.mex.2022.101876

**Published:** 2022-10-14

**Authors:** Adam R. Jensen, Ioannis Sifnaios, Kevin Anderson

**Affiliations:** a Department of Civil and Mechanical Engineering, Technical University of Denmark, Denmark; b National Renewable Energy Laboratory, USA

**Keywords:** Concentrated solar power (CSP), Concentrating photovoltaics (CPV), Concentrating solar collector, Dual axis tracker (DAT), Mutual shading, Open source, Python package

## Abstract

Self-shading in fields of two-axis tracking collectors typically ranges from 1% to 6% of the annual incident irradiation. It is thus essential to account for shading in order to obtain accurate yield estimates and financing for such solar projects. The present study presents the free and open-source Python package *twoaxistracking* for simulating self-shading in fields of two-axis tracking collectors. The package is freely available at: https://github.com/pvlib/twoaxistracking. The main steps of the method and mathematical formulation are described. Additionally, a demonstration of how to use the package is presented. The shading calculation method excels over previous methods found in the literature in that it can:

• Handle arbitrary aperture geometries and distinguish between the total and active areas.
• Account for sloped ground and collectors with different heights within the same field.
• Reduce computation time by skipping calculations at high solar elevation angles.

## Graphical abstract

**Figure:**
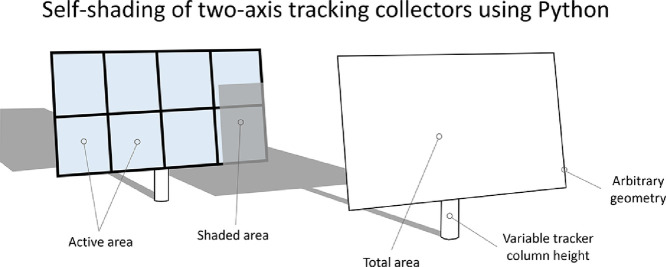

Specifications table

**Table:** 

Subject Area:	Energy
More specific subject area:	Solar energy
Method name:	Analytical calculation of self-shading of two-axis tracking collectors
Name and reference of original method:	Analytically Calculating Shading in Regular Arrays of Sun-Pointing Collectors [1] Self-shading of two-axis tracking solar collectors: Impact of field layout, latitude, and aperture shape [3]
Resource availability:	The documentation is available at: https://twoaxistracking.readthedocs.io The source code is available on GitHub: https://github.com/pvlib/twoaxistracking

## Method details

Two-axis trackers are typically used with highly concentrating solar energy technologies due to their ability to always orient the collecting surface normal to the sun. However, two-axis trackers typically experience greater shading losses than fixed-tilt collectors [2]. Failing to account for shading by neighboring collectors, referred to as self-shading, results in an overestimation of power production at low solar elevation angles. Typical annual shading losses are between 1% for sparse field layouts and 6% for dense field layouts [3]. As accurate yield estimations are critical to obtaining financing for solar energy projects, it is imperative that validated methods are available for accounting for shading.

## Background

Fixed-tilt and one-axis trackers can often be assumed to be of infinite length for modeling purposes, allowing shading to be modeled as one-dimensional [4]. Consequently, it is possible to derive simple analytical equations for calculating the shaded fraction. However, modeling self-shading of two-axis trackers is significantly more complex and is typically determined iteratively as multiple collectors can shade the same collector simultaneously. The term collector is used throughout this paper to denote either solar thermal collectors or photovoltaic panels.

One of the first methods for simulating shading of two-axis tracking collectors was proposed by Apley in [5]. The method approximates the collector surfaces as a grid of discrete points and calculates the shaded fraction of a reference collector as the ratio of shaded points to the total number of points. Discrete methods, such as the one proposed in [5], have the drawback that their accuracy depends on the number of points used. To overcome this limitation, Linn and Zimmerman [6] developed an analytical method suitable for trackers with circular and rectangular apertures. This method calculates the shaded fraction by identifying different cases of overlapping shading and uses separate equations for each case. However, the provided shading cases by Linn and Zimmerman only account for shading by immediate neighboring collectors. Subsequently, Meller [1] proposed a method for analytically calculating shading for arbitrary geometries. Regrettably, Meller only implemented the method for trackers with circular apertures and did not disclose the source code. To provide the scientific community with a validated open-source model, a new method was developed in [3] and implemented in Python.

The present study describes an extension of the validated method developed in [3] and provides an implementation in a free and open-source Python package called *twoaxistracking*. The presented method extends the basic shading calculations by:

• Differentiating between the total and active collector areas.
• Accounting for sloped fields and trackers with different tracker column heights.
• Reducing calculation time by skipping shading calculation for high solar elevations where shading is not possible.

To make the modeling process more user-friendly, the field layout and shading functions were implemented in a class (a type of data structure). Furthermore, the package is part of an established and growing community of free and open-source Python packages and is designed to be easily integrated with existing tools. Version 0.2.2 of the *twoaxistracking* package is described in this paper.

The remainder of this paper is structured according to the three main steps: (1) collector definition, (2) field layout, and (3) shading calculation. Lastly, the shading calculation method is validated and a short description of how to install the package is provided.

## Collector definition

The first part of the process is to define the collector geometry as the combination of two components:

• Total geometry: the overall gross area of the collector, including the frame. This is the geometry that *casts* shadows onto neighboring collectors.
• Active geometry: the area of the collector that contributes to power generation. This is the geometry for which *received* shadows from neighboring collectors are relevant.

**Fig. 1:**
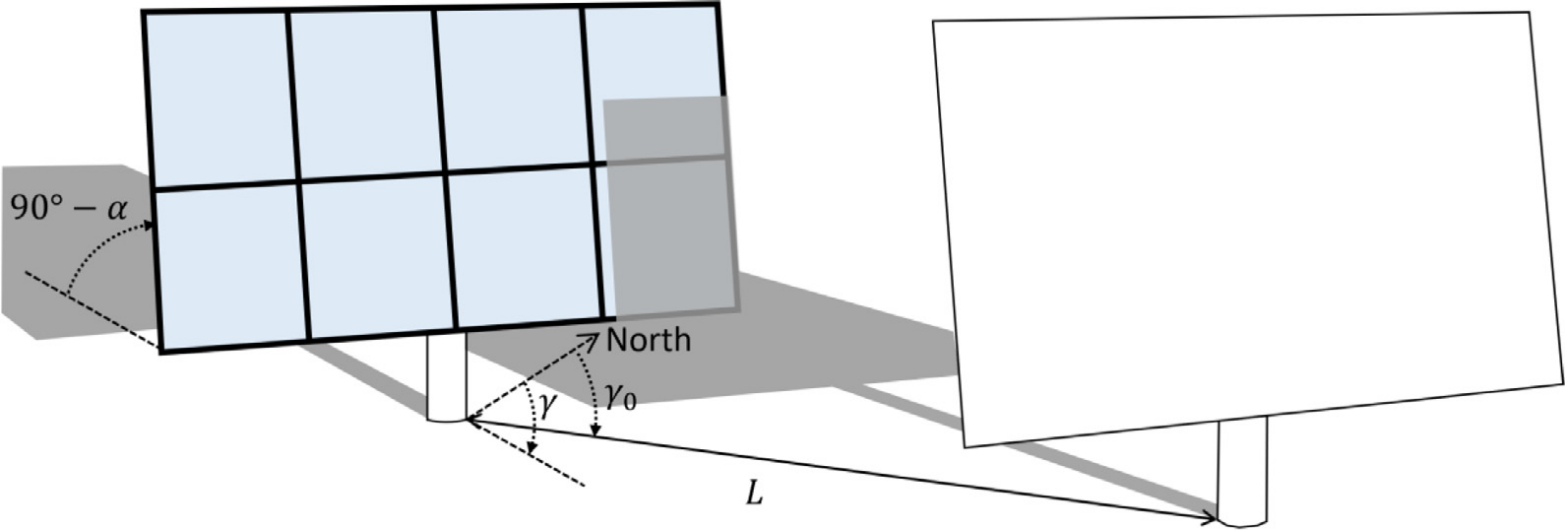
Illustration of a reference collector being shaded by a neighboring collector. The active area of the reference collector is shown in blue, whereas the total area comprises both the black and blue areas. The black area corresponds to the frames and mounting supports.

Although the total geometry determines the casted shadow, only the portion of the shadow covering the active area has an effect on power generation. Therefore, the shaded fraction is defined here as the shaded fraction of the active area rather than the shaded fraction of the total area. Note that differentiating between total and active area is an improvement over existing methods. An example of the difference between total and active areas is illustrated in Fig. 1 based on the geometry of the Fresnel lens concentrating solar collectors in Lendemarke, Denmark [7].

Due to the two-axis tracker motion, the collector surface is always maintained normal to the sun; thus, the collector tilt is equal to the complement of the solar elevation angle, and the collector azimuth is the same as the solar azimuth. The solar elevation, *α*, measured upwards from the horizon, and the solar azimuth, *γ*, measured clockwise from north, are shown in Fig. 1. The position of the neighboring tracker is defined by the distance, *L*, and the relative azimuth, *γ*_0_, to the reference collector.

The collector geometries are defined using the computational geometry Python package Shapely [8]. The total collector area is defined as a single polygon, whereas the active area can be described by one or more polygons (e.g., representing multiple panels or lenses). A single polygon is an instance of the Shapely Polygon class, whereas multiple polygons are of the Shapely MultiPolygon class.

**Fig. 2:**
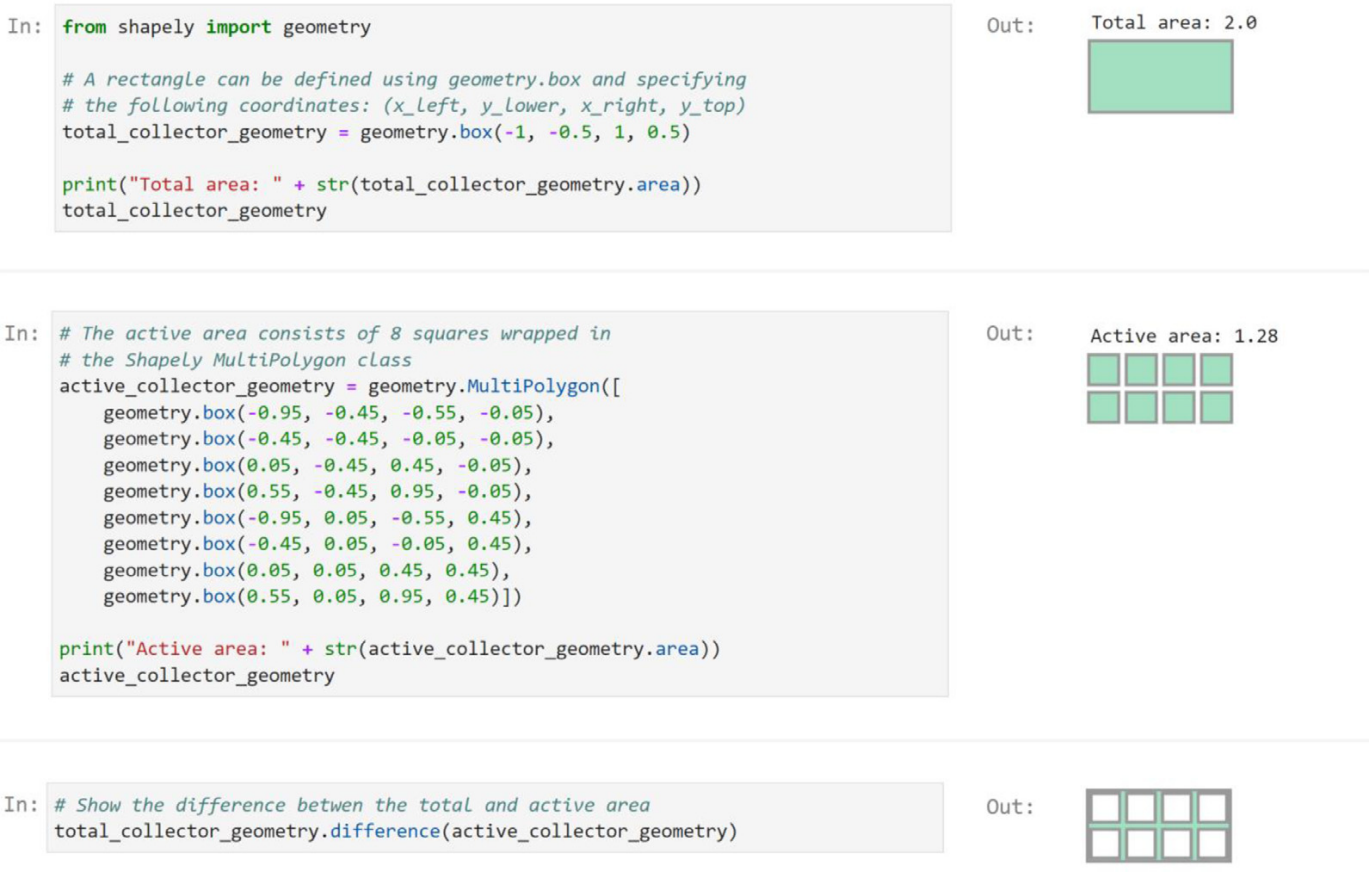
Example of how to define the total and active collector geometries. The total collector area is represented by a rectangle with an area of 2, whereas the active collector geometry consists of eight squares with a combined area of 1.28.

The total collector area is always greater than or equal to the active area, and the total collector geometry should completely enclose all the active areas. An example of how to define the total and active geometries is demonstrated in Fig. 2 (the geometry approximates the collector shown in Fig. 1). As shown in Fig. 2, each polygon is defined by a set of vertices specified with respect to the origin. For example, the total collector geometry is defined as a rectangle by specifying the lower-left coordinate (–1, –0.5) and the top-right coordinate (1, 0.5). Similarly, the active area consists of eight squares 0.4 by 0.4. The difference between the total and active area corresponds to a frame around the active areas with a width of 0.05.

The origin is assumed to be the center of rotation; thus, it is possible to simulate a collector that does not rotate around the center of the collector aperture. This is an important distinction, as it influences how close neighboring collectors can be placed, i.e., the further towards the edge of the collector aperture the rotation point is, the further a collector extends from the tracker column. This increases the distance that a neighboring collector must be placed, decreasing the maximum feasible ground coverage ratio (GCR). The ground coverage ratio is defined as the ratio between the total collector area and the total field area. The minimum spacing between two collectors can be calculated as twice the distance from the center of rotation (origin) to the furthest point of the collector aperture (total collector geometry). This distance is also known as the Hausdorff distance and ensures that two neighboring collectors cannot collide. It should be noted that any length unit can be used to define the collector geometries, though it is important to be consistent.

## Field layout

The following section describes the field layout generation and the associated parameters. According to Pons and Dugan [9], for fields with more than 50 collectors, it can reasonably be assumed that all collectors experience the same shading as a collector deep within the field. This indicates that edge effects can be ignored, and thus, the shaded fraction only has to be calculated for one reference collector. Nevertheless, it is necessary to decide how many neighboring collectors to consider in the shading calculations, which is configurable using the neighbor order parameter.

**Fig. 3:**
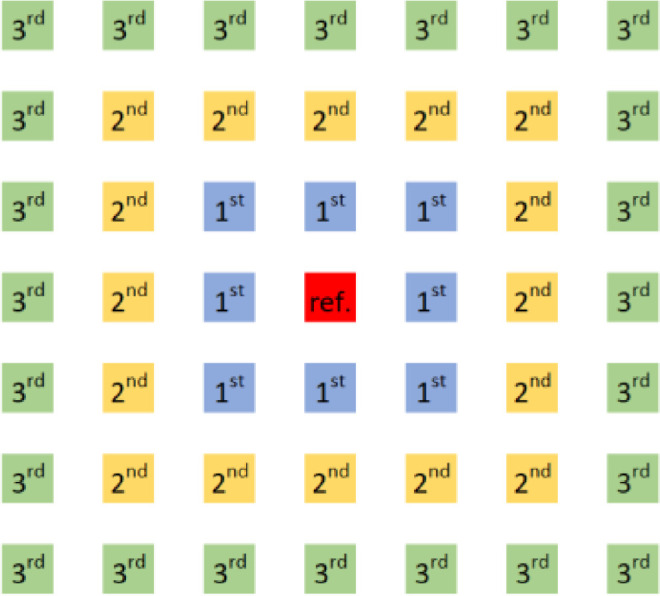
Graphical illustration of neighbor order. The reference collector is shown in red in the center, and the neighboring collectors are marked with the number corresponding to the lowest neighbor order for which they are considered.

A neighbor order of one corresponds to considering only the immediate neighboring collectors (see Fig. 3). For a neighbor order of two, the collectors directly adjacent to the first order neighbors are also considered, increasing the number of considered neighboring collectors from 8 to 24. The further away a neighboring collector is, the less effect it has on shading of the reference collector. As computation time increases drastically with increasing neighbor order, most studies use either a neighbor order of one or two. The authors recommend using a neighbor order of two, although the presented method can simulate any neighbor order.

**Fig. 4:**
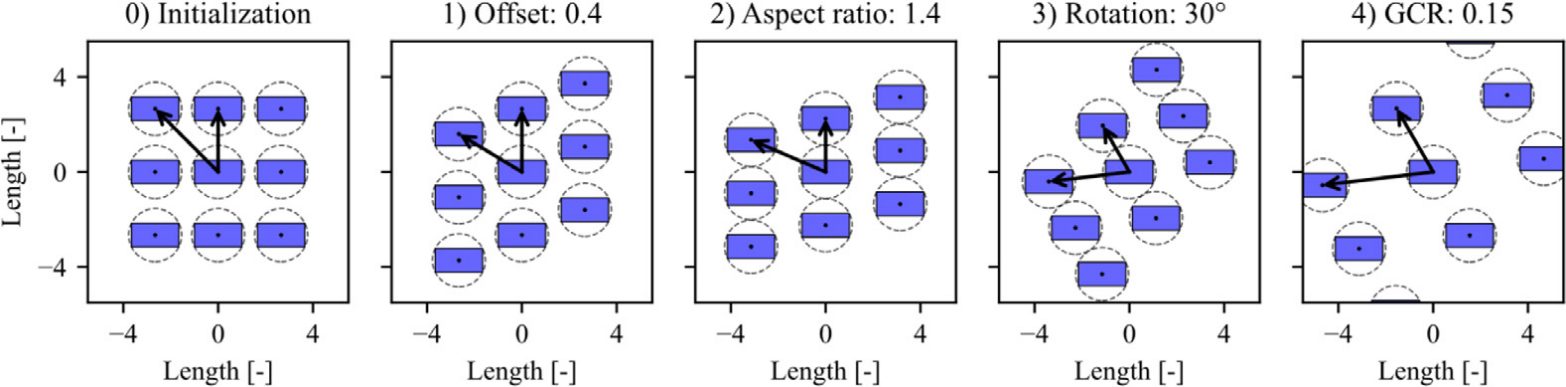
Illustration of the defining field layout parameters applied in succession, ending with a fully defined field layout [3]. The illustrated field layout has a neighbor order of one, and the dashed circles represent the minimum tracker spacing.

The field layout generation described in this paper is suitable for all regular field layouts, which encompasses almost all layouts investigated in the literature. A regular field layout is defined as a field layout where all collectors have the same arrangement of immediate neighbors. Any regular field layout can be defined by four parameters: offset, aspect ratio, rotation, and ground coverage ratio (illustrated in Fig. 4). The parameters are defined in [10]:

• Row offset: The relative offset of adjacent collector columns as a fraction of the distance between collector rows.
• Aspect ratio: The ratio of the distance between collector columns to the spacing between collector rows.
• Rotation: Counterclockwise rotation of the collector field.
• Ground coverage ratio (GCR): The ratio of the total collector area to the area of ground reserved by each collector.

The field layout is generated by applying a sequence of transformations to a basic layout where the reference collector is located at the center (0,0). The initial basic layout is defined by *X* and *Y*, representing the set of coordinate pairs (*x, y*) where *x* and *y* are integers and (*x, y*) lie within a square centered on (0, 0) with side length *N*. The side length can be calculated as:

(1) N=2·neighbor_order+1

Once the basic layout has been created, the four defining field layout parameters are applied in succession, as shown in Fig. 4. Eq. (2) through Eq. (5) correspond to the sequence of transformations applied in the code. Note that some of the transformations only affect either the *x* or *y* coordinates.

First, the offset is applied:

(2) Y→Y+offset·X

Second, the effect of the aspect ratio is accounted for:

(3) X→X·aspect_ratio

Next, the rotation is applied (counterclockwise):

(4) $$\left[\begin{array}{c} X\\ Y \end{array}\right]\to \left[\begin{array}{cc} \cos\left(-rotation\right) & \sin\left(-rotation\right)\\ -\sin\left(-rotation\right) & \text{cos}\left(-rotation\right) \end{array}\right]\left[\begin{array}{c} X\\ Y \end{array}\right]$$

Finally, the tracker positions are scaled in order to achieve the specified ground coverage ratio:

(5) $$\begin{array}{c} X\to X\cdot scaling\_factor\\ Y\to Y\cdot scaling\_factor \end{array}$$

where the scaling factor is defined as:

(6) $$scaling\_factor=\sqrt{\frac{total\_collector\_area}{gcr\cdot aspect\_ratio}}$$

**Fig. 5:**
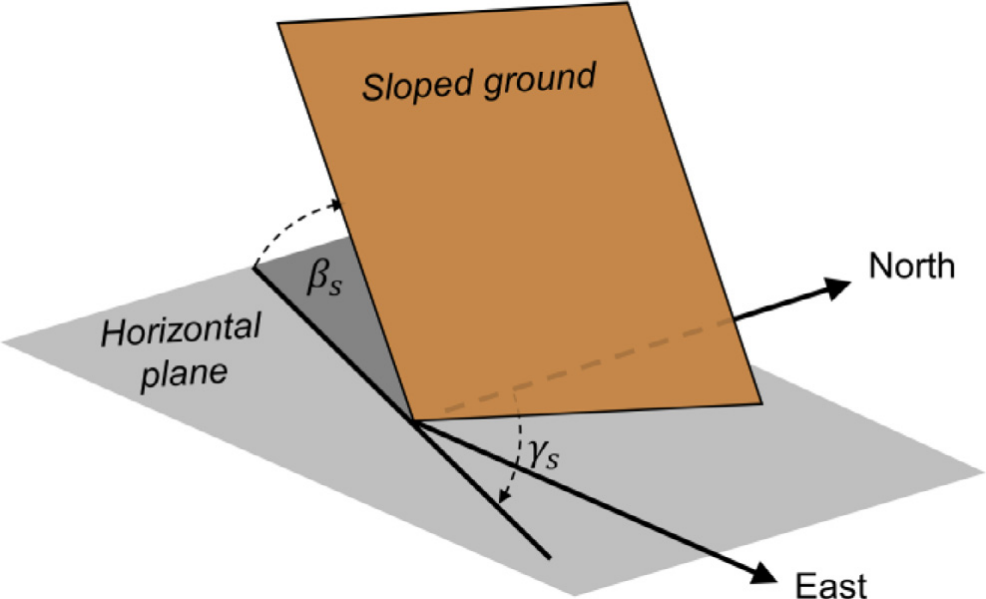
Illustration of the field slope azimuth and tilt angle. The sloped field plane is shown in brown.

Eq. (2) through Eq. (6) can be used to generate any regular field layout but do not account for a potential slope of the tracker field. In the present study, a sloped field is defined using the parameters slope azimuth and slope tilt. The slope azimuth, *γ_s_*, represents the direction of the normal to the slope when projected on the horizontal, i.e., in the direction of falling slope. The slope tilt, *β_s_*, is the angle of the sloped plane relative to the horizontal and is always positive. The slope azimuth and tilt angles are illustrated in Fig. 5.

To account for the slope in the field layout, an additional coordinate is required. To this end, the height of the neighboring collector relative to the reference collector is defined by the z coordinate, which is normal to the x-y plane. The corresponding set of z coordinates, Z, can be calculated as:

(7) $$Z=-X\cdot \sin\left(\gamma_{s}\right)\cdot \tan\left(\beta_{s}\right)-Y\cdot \cos\left(\gamma_{s}\right)\cdot \text{tan}\left(\beta_{s}\right)$$

Note, the x and y coordinates are independent of the slope, as the GCR is with respect to the vertical projection of the land area. This definition is the most common in the literature.

Furthermore, while X, Y, and Z completely define the layout, it is helpful to represent the collector positions in a spherical coordinate system as this greatly simplifies the shading calculations. Instead of the x and y coordinates, the horizontal collector position can be represented by the distance to the reference collector and the relative azimuth of the neighboring collector relative to the reference collector (illustrated in Fig. 1). The distance between a neighboring tracker and the reference tracker, L, is defined in Eq. (8).

(8) $$L=\sqrt{x^{2}+y^{2}}$$

The relative azimuth, $\gamma_{0}$, is defined in Eq. (9) and uses the modulus operation in order for the angle to be specified counterclockwise from north as shown in Fig. 1.

(9) $$\gamma_{0}=\left(450-\arctan2\left(Y,\;X\right)\right)\;\text{mod}\;360$$

Instead of the z coordinate, the height of the collectors can also be represented by the slope of the line between the reference and neighboring trackers. The relative slope, $\beta_{r}$, is positive when a neighboring collector is higher than the reference collector (z>0).

(10) $$\beta_{r}=\arctan\left(-\cos\left(\gamma_{s}-\gamma_{0}\right)\cdot \tan\left(\beta_{s}\right)\right)$$

The derivation of the relative slope is similar to the cross-slope axis tilt described in [11].

**Fig. 6:**
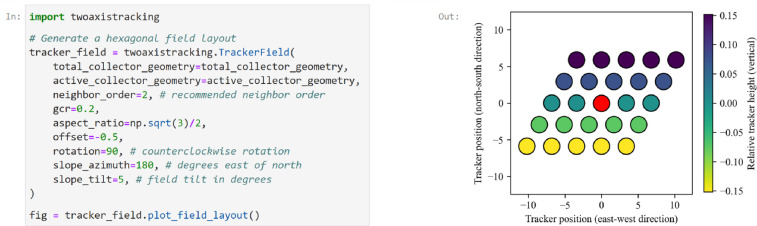
Demonstration of how to use the TwoAxisTrackerField class to generate a field layout. The figure on the right shows the reference collector in red, and the neighboring collectors are colored according to the relative tracker height (***z***).

The field layout can be conveniently specified using the class TwoAxisTrackerField, which provides a container for the collector geometry and the field layout. This considerably simplifies the modeling procedure and avoids users having to manually pass variables from one function to the next. The class also pre-calculates several properties, including the minimum tracker spacing, and provides a user-friendly method for calculating the shaded fraction. Defining and plotting a field layout using the TwoAxisTrackerField class is demonstrated in Fig. 6. Specifically, the example shows the definition of a hexagonal field layout with a ground coverage ratio of 0.2, a 5° slope towards the south, and is based on the collector geometries specified in Fig. 2.

Although regular layouts described by the four field layout parameters cover most typical use cases, the *twoaxistracking* package can also be used with custom collector positions corresponding to irregular field layouts. This could be useful for cases such as studying edge effects or simulating trackers with different heights. In that case, the users must explicitly specify the collector positions themselves.

## Shading calculation

The shading calculation method follows the general procedure described in [3], with the main difference being that the equations are modified to account for differences in tracker heights. The method accounts for shading iteratively, i.e., the shadow cast by each neighboring collector is assessed one at a time. As the shaded fraction differs with tracker position, shading has to be calculated for each unique solar position. At the start of each calculation, the unshaded geometry is initialized as the full active collector geometry. Then, for each neighboring collector, the shadow cast on the reference collector is determined and subtracted from the unshaded geometry. Overlapping shading is thus accounted for, as the shade cast on the same part of the reference collector by two different neighboring collectors can only be subtracted once.

**Fig. 7:**
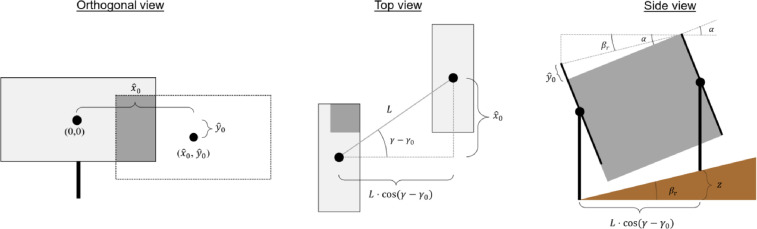
Illustration of a reference collector shaded by a neighboring collector on a sloped surface.

The central part of the shading calculation is to determine what part of the reference collector is shaded by each neighboring collector. To do this, the shadow of the shading collector is projected onto the plane of the reference collector (see Fig. 7). As the collector planes are parallel, the projected shadow has the same shape as the total collector geometry but is offset relative to the reference collector. The offset can be described by the shift in the $\hat{x}$ and $\hat{y}$-directions (${\hat{x}}_{0}$ and ${\hat{y}}_{0})$ as illustrated in the orthogonal view in Fig. 7. The $\hat{x}$- and $\hat{y}$-axes define a Cartesian coordinate system in the collector plane, where $\hat{x}$ is horizontal (from the sun's point of view), and $\hat{y}$ is perpendicular to $\hat{x}$ and points up the collector's slant height.

The offset can be calculated geometrically based on the sun position and the position of the specific neighboring collector. The offset in the $\hat{x}$-direction can be derived using the angles and distances shown in the top view in Fig. 7:

(11) $${\hat{x}}_{0}=L\cdot \text{sin}\left(\gamma -\gamma_{0}\right)$$

Notice that the offset in the $\hat{x}$-direction is not affected by the field slope or tracker height.

The offset in the $\hat{y}$-direction can be derived based on the side view schematic in Fig. 7:

(12) $${\hat{y}}_{0}=-\sin\left(\alpha -\beta_{r}\right)\cdot \frac{L\cdot \cos\left(\gamma -\gamma_{0}\right)}{\cos\left(\beta_{r}\right)}$$

**Fig. 8:**
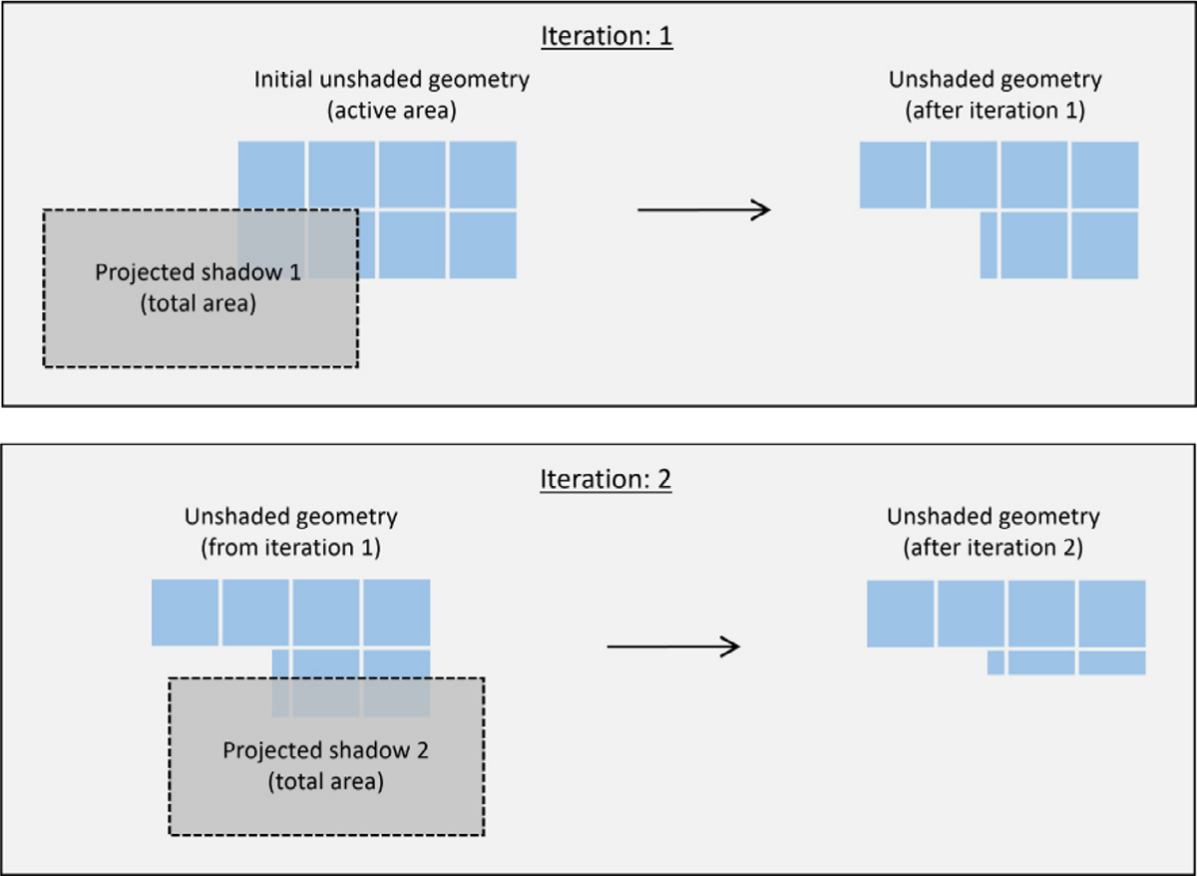
Illustration of the iterative procedure of determining the unshaded area. View is orthogonal to the collector plane.

Once the offsets have been determined, the additional shaded area of the reference collector can be calculated as the intersection of the unshaded geometry and the projected shadow. This calculation is executed using the Shapely *difference* function. The remaining unshaded geometry is then used for the next iteration, i.e., the next neighboring collector. The iterative procedure is illustrated in Fig. 8. Once this procedure has been repeated for all neighboring collectors under consideration, the unshaded area is calculated.

**Fig. 9:**
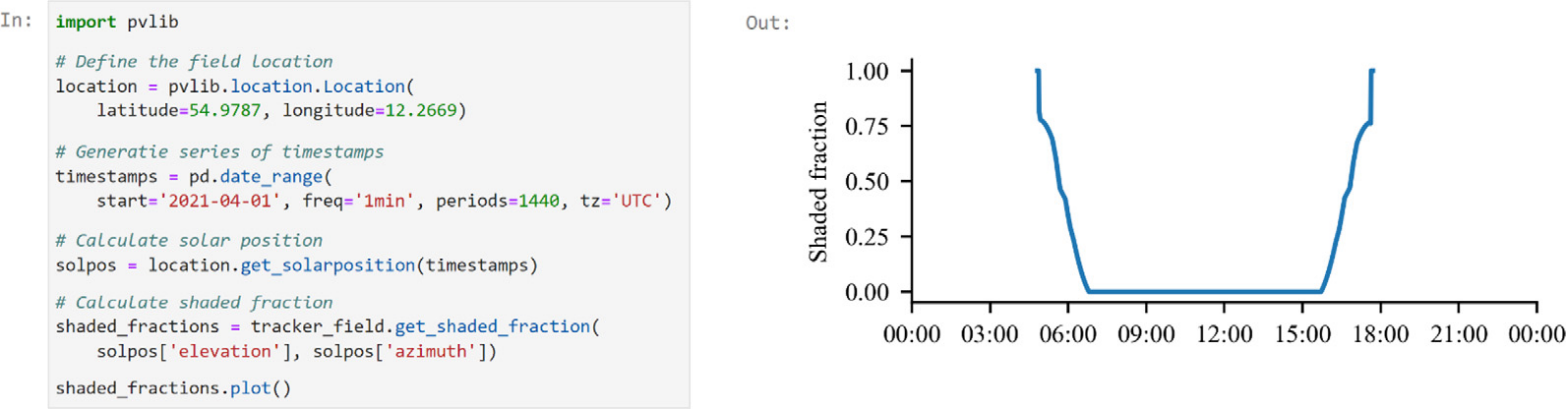
Demonstration of how to calculate the shaded fraction for one day. The calculations are based on the collector defined in Fig. 2 and the field layout shown in Fig. 6.

The TwoAxisTrackerField class wraps the shading calculation function; thus, to calculate the shaded fraction, the user only needs to specify a list of solar elevation and azimuth angles. The calculation of the solar position and shaded fraction for one day is demonstrated in Fig. 9. The solar position is calculated for Lendemarke, Denmark, using the pvlib-python Python package [12].

## Selection of possible shading collectors

The shading calculations should only take into account neighboring collectors that are within a field of view ±90°, i.e., in front of the reference collector. It is desirable to reduce the number of considered collectors further to decrease the computational time. As described in [1], if the bounding circles of the reference collector and the projected shadow do not overlap, shading cannot occur, and the collector can be left out of the shading calculation for that time step. This criterion can be expressed as:

(13) $$\sqrt{{\hat{x}}_{0}^{2}+{\hat{y}}_{0}^{2}}<D_{min}$$

where $D_{min}$ is the diameter of the bounding circle. In the present study, the minimum collector spacing is used as an approximation of the bounding circle diameter. This assumption is exact for collectors that rotate around their center and is a conservative estimate for collectors that do not.

## Maximum shading elevation

The *twoaxistracking* package also introduces a new strategy for reducing the shading calculation time by skipping calculations for which the solar elevation angle is above the maximum threshold. The maximum shading angle is the highest angle for which shading can occur for a given collector geometry and field layout. For sparse collector fields (GCR < 0.2), this can result in a simulation time reduction between 15 and 35%. However, for dense field layouts (GCR > 0.4), there is only a minor to no reduction in calculation time.

The maximum shading elevation can be calculated analytically for rectangular and circular collectors. Collectors that do not fit into these two categories can be approximated either by using the bounding box of the collector geometry or the bounding circle. For a rectangular collector aperture, the maximum shading elevation occurs when ${\hat{x}}_{0}$ is equal to the width of the collector and $-{\hat{y}}_{0}$ is equal to the collector height. For a circular collector, the maximum shading elevation occurs when ${\hat{x}}_{0}$ is zero and $-{\hat{y}}_{0}$ is equal to the diameter, i.e., the projected shadow is directly below the reference collector. In either case, the maximum elevation angle can then be calculated using Eqs. (11) and (12) and solving for α. To ensure that the lowest maximum shading elevation is found, both methods are applied, and the lowest shading elevation is chosen.

## Horizon shading

Furthermore, when modeling sloped fields, it is important to consider the blockage of the sun caused by the sloped field. For example, for a field with a 5° slope towards the south, the sun is blocked for solar elevation angles less than 5° towards the north. The horizon angle caused by a sloped hill can be calculated for each azimuth angle:

(14) $$horizon\_angle\left(\gamma \right)=\;\text{max}\left(\arctan\left(-\cos\left(\gamma_{s}-\gamma \right)\cdot \tan\left(\beta_{s}\right)\right),\;0\right)$$

**Fig. 10:**
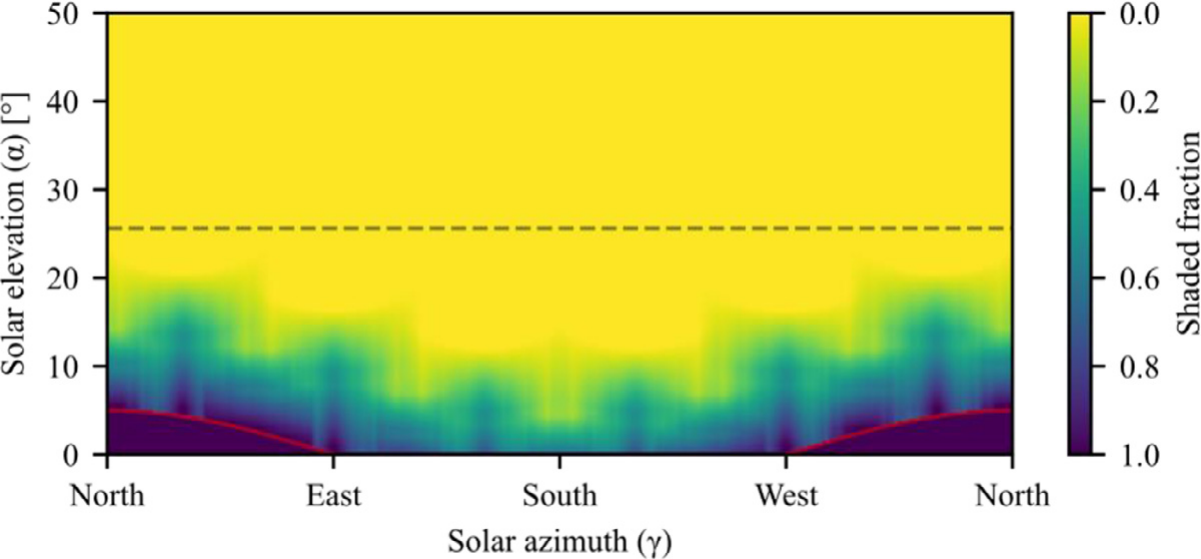
Shaded fraction as a function of solar elevation and azimuth angles for the field layout shown in Fig. 6. The solid red line indicates the horizon line caused by the sloped field, and the dashed black line indicates the maximum elevation for which shading can occur.

When the solar elevation angle is below the horizon line, the shaded fraction is set to zero, and the shading calculations are skipped. The shaded fraction for the field layout shown in Fig. 6 is shown as a function of solar elevation and azimuth angle in Fig. 10.

## Validation

To validate the shading calculation method, the annual shading loss (ASL) was calculated for the same nine field layouts reported in [10]. Cumpston and Pye [10] utilized the shading calculation algorithm developed by Meller [1], which has previously been compared to [9]. The simulations were carried out using the Barstow 1976 reference year, and the calculated annual shading losses are presented in Table 1. The annual shading loss is defined as the fraction of direct normal irradiation lost due to shading relative to the incident direct irradiation when no shading occurs. The differences between the ASL reported in [10], and the values derived using the method presented in this work (ASL *twoaxistracking*) are very small, with a maximum absolute difference of 0.2% and a maximum relative difference of 1.3%. As expected, these values are nearly identical to the values reported in [3], as the present method is an extension of the method presented in [3]. The minor discrepancies between the annual shading losses are believed to be due to differences in the quality control procedure applied to the irradiance dataset, which was necessary to remove erroneous periods.

**Table 1:** Comparison of the annual shading loss (ASL) from the reference study [10] and calculated by the method described in this paper for nine different field layouts [3]. See [10] for the specific field layout parameters. All values are in percentages.

GCR	ASL [10]	ASL *twoaxistracking*	Absolute deviation	Relative deviation
0.1	0.04	0.04	0.00	0.00
0.2	1.53	1.51	−0.02	−1.31
0.3	4.50	4.49	−0.01	−0.22
0.4	8.14	8.09	−0.05	−0.61
0.5	12.4	12.3	−0.10	−0.81
0.6	17.2	17.1	−0.10	−0.58
0.7	22.3	22.2	−0.10	−0.45
0.8	27.6	27.4	−0.20	−0.72
0.9	33.1	32.9	−0.20	−0.60

## Conclusion

This work presented the theoretical basis for analytically calculating self-shading in fields of two-axis trackers. The presented method is a significant advancement compared to existing methods in that it is able to account for differences in the active and total area, model fields with sloped ground, and implements a novel algorithm for reducing the computation time required. The method has been validated and implemented in a free and open-source python package called *twoaxistracking*, which makes it easy for users to calculate shading for their own applications.

## Installation

The package is available at the Python Package Index (PyPI) and can be installed with the following command:

Alternatively, the source code can be downloaded from the project's GitHub repository and installed manually.

## Data Availability

No data was used for the research described in the article.
